# Upshot of heterogeneous catalysis in a nanofluid flow over a rotating disk with slip effects and Entropy optimization analysis

**DOI:** 10.1038/s41598-020-80553-1

**Published:** 2021-01-08

**Authors:** Muhammad Ramzan, Saima Riasat, Jae Dong Chung, Yu-Ming Chu, M. Sheikholeslami, Seifedine Kadry, Fares Howari

**Affiliations:** 1 Department of Computer Science, Bahria University, Islamabad, 44000 Pakistan; 2 Department of Mechanical Engineering, Sejong University, Seoul, 143-747 Korea; 3 Department of Mathematics, Huzhou University, Huzhou, 313000 People’s Republic of China; 4 Hunan Provincial Key Laboratory of Mathematical Modeling and Analysis in Engineering, Changsha University of Science and Technology, Changsha, 410114 People’s Republic of China; 5 Department of Mechanical Engineering, Babol Noshirvani University of Technology, Babol, Iran; 6 Renewable Energy Systems and Nanofluid Applications in Heat Transfer Laboratory, Babol Noshirvani University of Technology, Babol, Iran; 7 Department of Mathematics and Computer Science, Faculty of Science, Beirut Arab University, Beirut, 115020 Lebanon; 8 College of Natural and Health Sciences, Zayed University, 144543 Abu Dhabi, United Arab Emirates

**Keywords:** Software, Mechanical engineering

## Abstract

The present study examines homogeneous (HOM)–heterogeneous (HET) reaction in magnetohydrodynamic flow through a porous media on the surface of a rotating disk. Preceding investigations mainly concentrated on the catalysis for the rotating disk; we modeled the impact of HET catalysis in a permeable media over a rotating disk with slip condition at the boundary. The HOM reaction is followed by isothermal cubic autocatalysis, however, the HET reactions occur on the surface governed by first-order kinetics. Additionally, entropy minimization analysis is also conducted for the envisioned mathematical model. The similarity transformations are employed to convert the envisaged model into a non-dimensional form. The system of the modeled problem with ordinary differential equations is analyzed numerically by using MATLAB built-in bvp4c function. The behavior of the emerging parameters versus the thermal, concentration, and velocity distributions are depicted graphically with requisite discussion abiding the thumb rules. It is learned that the rate of the surface catalyzed reaction is strengthened if the interfacial area of the permeable media is enhanced. Thus, a spongy medium can significantly curtail the reaction time. It is also noticed that the amplitude of velocity and thermal profile is maximum for the smallest value of the velocity slip parameter. Heat transfer rate declines for thermophoresis and the Brownian motion parameter with respect to the thermal slip parameter. The cogency of the developed model is also validated by making a comparison of the existing results with a published article under some constraints. Excellent harmony between the two results is noted.

## Introduction

The nanofluids found abundant industrial and engineering applications owing to their exceptional thermal performance in comparison to ordinary fluids like water. Nanofluids are composed of nanometer-sized particles immersed into the base fluids. The mixture of these nanoparticles into the base liquid in a specific ratio possesses excellent thermal performance and is being widely used for several years in many industrial and engineering processes. Choi and Eastman^1^ were the pioneers who devoted their attention to nanofluid applications in various areas of science and technology. The promising thermophysical characteristics of nanofluids has motivated the researchers to profuse theoretical and experimental studies. Eastman et al.^2^ worked out that ethylene glycol-based nanofluid with copper (Cu) as nanoparticles has much better thermal conductivity rather than simple ethylene glycol or ethylene glycol with dispersed oxides of nanoparticles. Buongiorno^3^ studied characteristics of nanofluids such as Brownian motion and thermophoresis to develop the model equations for the mass, momentum, and heat transfer. Tiwari and Das^4^ erected the model to evaluate the thermal conductivity by considering the solid volume fraction in the heated square cavity. Some important investigations highlighting the role of nanofluids in varied capacities may be found in^5–10^ and many therein.

The study of thermophysical characteristics of nanofluid flow past a rotating disk has motivated many researchers and scientists for its applications in industry such as flywheels, rotors, shrink fits and gears, computer disk, electric power generation, rotor–stator spinning disc reactors, crystal growth process, etc. Initially, Karman^11^ studied the problem of rotating plane lamina. To investigate the physical phenomenon, the Von Karman transformation is used. Cochran^12^ extended the work of Karman to obtain a more accurate numerical solution to the problem. Stewartson^13^ analyzed the confined flow between rotating coaxial disks. Numerical treatment of the slip flow past a rotating disk for non-Newtonian fluid is studied by Naqvi et al.^14^. Waqas et al.^15^ contemplated the numerical investigation of bioconvective nanofluid flow past a rotating disk. Khan et al.^16^ examined the swirling chemical reactive nanofluid flow past a rotating disk. Abbas et al.^17^ explored the Oldroyd-B nanofluid flow over a rotating disk. Tlili et al.^18^ investigated the hybrid nanofluid flow by considering slip effects. Sheikholeslami et al.^19^ investigated the impact of magnetic force with a porous enclosure having an elliptic shape obstacle. behavior in a porous enclosure with an ellipse-shaped obstacle. Zaimi et al.^20^ performed a nanofluid flow analysis of over a nonlinear sheet. Ramzan et al.^21^ explored the entropy generation analysis of magnetized carbon nanotubes-based nanofluid flow. Shah et al.^22^ investigated the activation energy of radiative flow past a nonlinear stretched surface.

Researchers have extensively investigated the combination of porous media and nanofluids for its potential applications in the heat transfer phenomenon. Porous media consist of many tiny pores, that would provide more surface area which is found to be useful in industrial production. The nanofluid catalytic reactive flow through porous media has been studied theoretically as well as experimentally. Hunt et al.^23,24^ devoted his investigations to resolve convective-diffusive transport phenomena with a porous solid phase and catalytic surfaces with thick wall subjected to thermal loads. Further, Guthrie et al.^25,26^ reported a theoretical analysis of first-order catalytic chemical reactions on the internal surfaces of parallel plates. The impact of wall thickness on thermal performance has been considered. The exothermic catalytic process to examine the heat transfer phenomenon with an inclined magnetic field in the inner walls of porous microreactors is accomplished by Saeed et al.^27,28^. Alizadeh et al.^29^ investigated forced convection in the presence of catalytic surface impinging stagnation flows embedded in porous media. Gomari et al.^30^ observed impinging nanofluid flow in a cylinder embedding in porous media that the concentration of nanoparticles has a considerable modification on thermal and hydrodynamic boundary layers, which results in the variation of entropy generation and Nusselt number. Ullah et al.^31^ investigated the Darcy-Forchheimer flow of nanofluid by a rotating disk with partial slip effects.

Most chemical reacting systems are dependent on the Homogeneous–heterogeneous (HOM–HET) reactions. The presence of catalyst enhances the rate of a chemical reaction. HET reaction occurs on the catalytic surface while the HOM reaction occurs in the same phase. The study of the chemical reactions found the application in many areas including manufacturing of ceramics, food processing, polymer production, hydrometallurgical products, and equipment designing via chemical reactions, etc. Chaudhary and Merkin^32^ studied the HOM–HET reactions with isothermal cubic autocatalytic. Recently HOM–HET reaction in the disk problem has been studied copiously. Recently Doh et al.^33^ studied the nanofluid flow over a disk of variable thickness in the presence of HOM–HET reaction. Tiwari and Das's model is adopted here comprising a water–silver combination to form a nanofluid. The major outcome of this study is that both radial and tangential velocities are enhanced when disk thickness is improved. The Darcy–Forchheimer nanofluid flow in the presence of carbon nanotubes with convective boundary conditions with HOM–HET reactions is analyzed by Hayat et al.^34^. The solution of the presented model is found in the form of series by employing the Homotopy Analysis Method. Gholinia et al.^35^ explored the Powell Eyring magnetohydrodynamic nanofluid flow accompanying homogeneous–heterogeneous reactions with slip condition. The numerical solution of the problem is obtained using the 4th and 5th order RK Fehlberg method. It is gathered that the fluid temperature is improved when the thermophoresis parameter values are increased. Hayat et al.^36,37^ studied the ferrofluid flow accompanying HOM–HET reactions owing to a rotating disk. The combination of the water–Fe_3_O_4_ is considered here. The salient conclusion of the study is that fluid concentration is decreased large estimates of HOM-reactions and the opposite trend is seen for HET-reactions.

Entropy measures the rate of the disorder in a system and its surroundings. It is a physical phenomenon of heat transfer in the form of energy. As the transfer of heat causes the change in kinetic energy, and potential energy, etc. Bejan^38,39^ was a pioneer, who did the entropy generation analysis during heat losing process of fluid motion. Liu et al.^40^ worked out the irreversibility of MHD flow in the curved channel. Qayyum et al.^41^ researched the entropy generation analysis of the Williamson fluid flow amid two rotating disks moving with dissimilar rotation rates. The major outcome of this exploration is that the axial and radial velocities depict opposite behavior for large estimates of Weissenberg number. The entropy analysis revealed that the entropy of the system is significantly enhanced when radiation effects are strengthened. The flow of Sisko nanofluid with activation energy accompanying binary chemical reaction past a radially stretching disk with irreversibility analysis is studied by Ijaz et al.^42^. An analytic solution to the problem is obtained using a homotopic scheme. The Casson nanofluid flow containing MnFe_2_O_4_–water combination in a Darcy–Forchheimer porous media over a disk is studied by Shaw et al.^43^. The irreversibility analysis of the presented model is also conducted. An interesting outcome of this model is that the entropy generation is used in the brain function analysis. Some more interesting investigations highlighting nanofluid flow over a rotating disk are given at^33,44,45^.

In the study of the flow of fluids, it is assumed that the fluid velocities on the surface and adjacent to the wall are nearly the same. But a close look discloses that small slips arise at the solid–fluid intersection owing to volatility at the high-stress levels as in the extraction of polymers. These fluid slips affect the fluid movement at the surface. A good number of studies can be seen in the literature because of the interesting effects of slips on fluid motion. Awais et al.^46^ performed the numerical treatment of viscous nanofluid flow over a rotating disk with Navier slip, chemical reaction, and the magnetohydrodynamic. The key result of the presented model is that the radial velocity is decreased for Reiner-Rivlin's large values. The 3D magneto flow of the nanofluid comprising single-walled carbon nanotubes immersed into water over an extended rotating disk with velocity and thermal slips is investigated by Nasir et al.^47^. It is gathered here that the fluid velocity is highly affected by the strong magnetic field.

Earlier investigators primarily concentrated on surface catalysis; we developed the influence of heterogeneous catalysis in a permeable media over the rotating disk with slip condition at the boundary. This is a new concept in the rotating disk area and has not been discussed in the literature yet. Thus, the focus of the present study is to develop the model by taking the nanofluid flow embedded in porous media over the rotating disk with partial slip effects on the momentum and heat equation. HOM–HET isothermal cubic autocatalysis is employed with homogeneous porous media. Former explorations considered HET reaction at the wall surface. However, in our assumption, stretched rotating surfaces and porous media chemical reaction proceeds in the presence of the same catalyst. In this manner, HET reaction also befalls on the stretched disk surface, named as a surface-catalyzed reaction. The uniqueness of the present model as depicted by Table 1 is verified by comparing the present model with the published researches.

**Table 1:** Literature survey for uniqueness of the presented model.

Authors	Buongior-no model	Rotating disk	Slip effect	Porous media	HOM–HET reactions	HET catalysis	Entropy effect
Naqvi et al.^14^	Yes	Yes	Yes	No	No	No	No
Ullah et al.^31^	Yes	Yes	Yes	Yes	No	No	No
Gholinia et al.^35^	Yes	Yes	Yes	No	Yes	No	No
Hayat et al.^48^	Yes	Yes	No	No	Yes	No	No
Hayat et al.^49^	Yes	Yes	Yes	No	No	No	No
Present	Yes	Yes	Yes	Yes	Yes	Yes	Yes

This reaction is elaborated by^50^:

1 $$ Sk_{s} C_{a} , $$

where $S$ denotes the area of the permeable medium. Kameswaran et al.^51^ investigated the nanofluid flow for homogeneous–heterogeneous reactions past a porous stretching sheet. Hayat et al.^36^ examined the stagnation point flow over the sheet of variable thickness.

## Problem formulation

**Figure 1:**
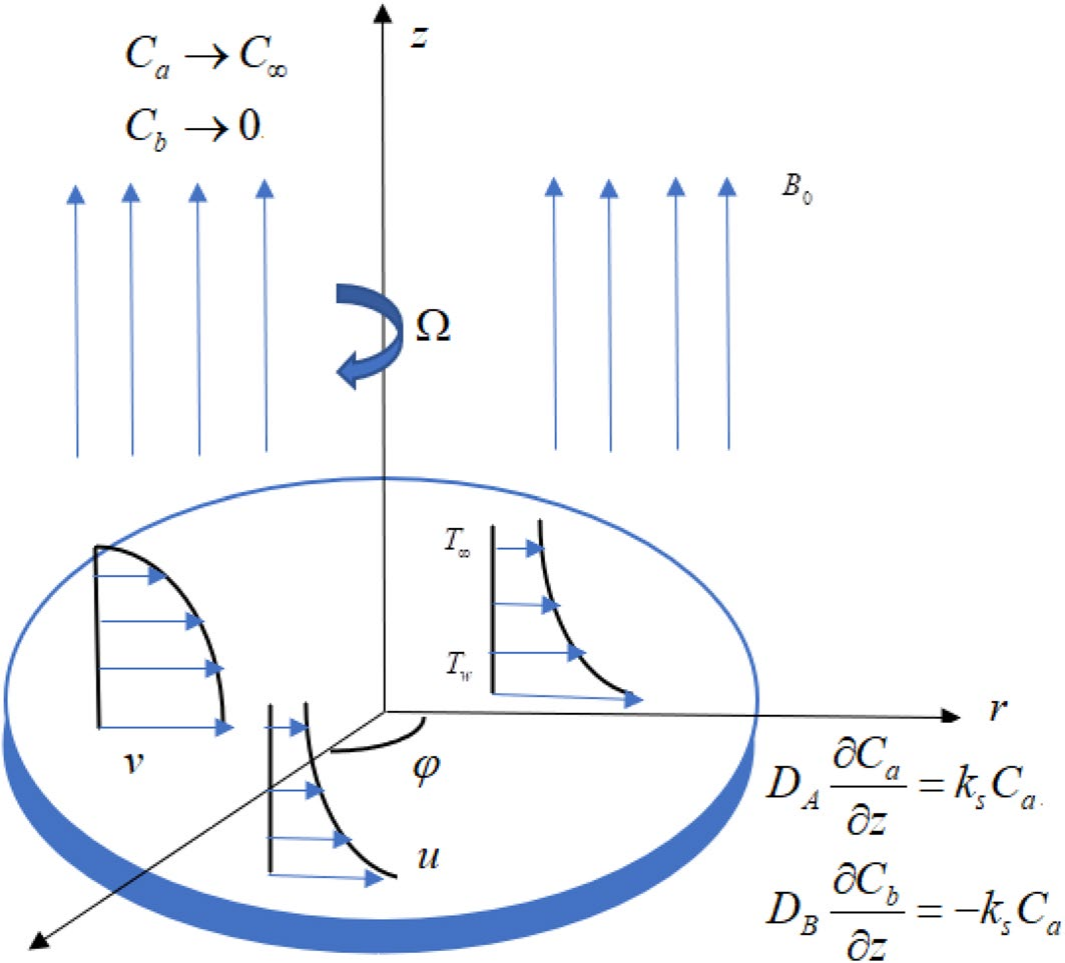
Geometrical sketch of the model problem.

Assume a three-dimensional flow of nanofluid past a disk having angular velocity $\Omega$ and the velocity & thermal slip conditions at the surface in the porous media. Viscous dissipation, Brownian motion, and thermophoresis effects are employed. The HOM–HET reactions are considered in the flow regime. The HOM reaction occurs in the fluid while the HET reaction occurs on the surface of porous media and disk (Fig. 1). Chaudhary and Merkin^32^ defined the process of isothermal cubic autocatalysis in the Nanofluid flow and is given by:

2 $$ A + 2B \to 3B. $$

HET reaction is given by:

3 $$ A \to B. $$

The HOM reaction rate is $k_{c} C_{a} C_{b}^{2}$ and the rate of HET reaction is $k_{s} C_{a}$. Where $C_{a}$ and $C_{b}$ are the concentration of chemical species $A$ and $B$ respectively. And the rate of reaction on porous media in porous media is given by $- Sk_{s} C_{a}$.

The governing system of equations under the above-mentioned assumptions is characterized by:

4 $$ \nabla .V = 0, $$

5 $$ (V.\nabla )u = \nu \left( {\nabla^{2} u} \right) - \frac{{\sigma B_{0}^{2} u}}{\rho } - \frac{\nu }{{k^{*} }}u - Fu^{2} , $$

6 $$ (V.\nabla )v = \nu \left( {\nabla^{2} v} \right) - \frac{{\sigma B_{0}^{2} v}}{\rho } - \frac{\nu }{{k^{*} }}v - Fv^{2} , $$

7 $$ \begin{gathered} (V.\nabla )T = \frac{k}{{\rho C_{P} }}\left( {\nabla^{2} T} \right) + \tau \left[ {D_{B} \left( {\frac{{\partial C_{b} }}{\partial r}\frac{\partial T}{{\partial r}} + \frac{{\partial C_{b} }}{\partial z}\frac{\partial T}{{\partial z}}} \right) + \frac{{D_{T} }}{{T_{\infty } }}\left( {\left( {\frac{\partial T}{{\partial r}}} \right)^{2} + \left( {\frac{\partial T}{{\partial z}}} \right)^{2} } \right)} \right] - \hfill \\ \frac{\mu }{{\rho C_{P} }}\left( {2\left( {\frac{\partial u}{{\partial r}}} \right)^{2} + 2\left( \frac{u}{r} \right)^{2} + 2\left( {\frac{\partial w}{{\partial z}}} \right)^{2} + \left( {\frac{\partial v}{{\partial z}}} \right)^{2} + \left( {\frac{\partial v}{{\partial r}} - \frac{v}{r}} \right)^{2} + \left( {\frac{\partial w}{{\partial r}} + \frac{\partial u}{{\partial z}}} \right)^{2} } \right), \hfill \\ \end{gathered} $$

8 $$ u\frac{{\partial C_{a} }}{\partial r} + w\frac{{\partial C_{a} }}{\partial z} = D_{A} \left( {\frac{{\partial^{2} C_{a} }}{{\partial r^{2} }} + \frac{1}{r}\frac{{\partial C_{a} }}{\partial r} + \frac{{\partial^{2} C_{a} }}{{\partial z^{2} }}} \right) - k_{c} C_{a} C_{b}^{2} - Sk_{s} C_{a} = 0, $$

9 $$ u\frac{{\partial C_{b} }}{\partial r} + w\frac{{\partial C_{b} }}{\partial z} = D_{B} \left( {\frac{{\partial^{2} C_{b} }}{{\partial r^{2} }} + \frac{1}{r}\frac{{\partial C_{b} }}{\partial r} + \frac{{\partial^{2} C_{b} }}{{\partial z^{2} }}} \right) + \frac{{D_{T} }}{{T_{\infty } }}\frac{{\partial^{2} T}}{{\partial z^{2} }} + k_{c} C_{a} C_{b}^{2} + Sk_{s} C_{a} = 0. $$

The boundary conditions are:

10 $$ \begin{gathered} u = L_{1} \frac{\partial u}{{\partial z}},v = r\Omega + L_{1} \frac{\partial v}{{\partial z}},w = 0,T = T_{w} + L_{2} \frac{\partial T}{{\partial z}}, \hfill \\ D_{A} \frac{{\partial C_{a} }}{\partial z} = k_{s} C_{a} ,D_{B} \frac{{\partial C_{b} }}{\partial z} = - k_{s} C_{a} ,\eta \to 0. \hfill \\ u \to 0,v \to 0,w = 0,T \to T_{\infty } ,C_{a} \to C_{\infty } ,C_{b} \to 0,\eta \to \infty . \hfill \\ \end{gathered} $$

Here, $L_{1}$ and $L_{2}$ are the velocity and temperature slip condition coefficients respectively. $F = \frac{{C_{b} }}{{r\sqrt {k^{*} } }}$ is the non-uniform inertia factor.

Applying the following similarity transformations:

11 $$ \begin{gathered} u = r\Omega f^{\prime}(\eta ),v = r\Omega g(\eta ),w = - \sqrt {\frac{2\Omega }{\nu }} f(\eta ),\eta = z\sqrt {\frac{2\Omega }{\nu }} , \hfill \\ \theta (\eta ) = \frac{{T - T_{\infty } }}{{T_{w} - T_{\infty } }},C_{a} = C_{\infty } \xi (\eta ),C_{b} = C_{\infty } \psi (\eta ), \hfill \\ \end{gathered} $$

The above partial differential equations take the subsequent form:

12 $$ 2f^{\prime\prime\prime} + 2ff^{\prime\prime} - f^{{\prime}{2}} + g^{2} + M^{2} f^{\prime} + \lambda f^{\prime} + F_{r} f^{{\prime}{2}} = 0, $$

13 $$ 2g^{\prime\prime} + 2fg^{\prime} - 2f^{\prime}g + M^{2} g + \lambda g + F_{r} g^{2} = 0, $$

14 $$ \frac{1}{\Pr }\theta^{\prime\prime} + f\theta^{\prime} + N_{b} \theta^{\prime} + N_{t} \theta^{{\prime}{2}} + Ec\left( {\frac{1}{{{\text{Re}}_{r} }}f^{{\prime}{2}} + g^{{\prime}{2}} + f^{{\prime\prime}{2}} } \right) = 0, $$

15 $$ \frac{1}{Sc}\xi^{\prime\prime} - K_{c} \xi \phi^{2} - K_{vs} \xi + f\xi^{\prime} = 0, $$

16 $$ \frac{\delta }{Sc}\left( {\phi^{\prime\prime} + \frac{{N_{t} }}{{N_{b} }}\theta^{\prime\prime}} \right) + K_{c} \xi \phi^{2} + K_{vs} \xi + f\phi^{\prime} = 0, $$

Assuming the diffusion coefficients of both species similar. $\delta = 1$, and $\xi (\eta ) + \phi (\eta ) = 1,$ which leads to the above Eqs. (15) and (16) to the following form:

17 $$ \frac{1}{Sc}\left( {\phi^{\prime\prime} - \frac{{N_{t} }}{{N_{b} }}\theta^{\prime\prime}} \right) - \phi K_{c} (1 - \phi )^{2} - K_{vs} \phi + f\phi^{\prime} = 0, $$

18 $$ \begin{gathered} f = 0,f^{\prime} = \gamma_{1} f^{\prime\prime},g = 1 + \gamma_{1} g^{\prime},\theta = 1 + \gamma_{2} \theta^{\prime},\phi^{\prime}(0) = \phi (0)K_{s} , \hfill \\ \phi (\infty ) = 1,f^{\prime}(\infty ) = 0,g(\infty ) = 0,\theta (\infty ) = 0. \hfill \\ \end{gathered} $$

The above ordinary differential equations involve dimensionless parameters including

19 $$ \begin{gathered} \lambda = \frac{\nu }{{k^{*} \Omega }},F_{r} = \frac{{C_{b} }}{{\sqrt {k^{*} } }},M^{2} = \frac{{\sigma B_{o}^{2} }}{\rho \Omega },\Pr = \frac{{\rho C_{p} \nu }}{k},N_{b} = \frac{{\tau D_{B} C_{\infty } }}{\nu }, \hfill \\ N_{t} = \frac{{\tau D_{T} (T_{w} - T_{\infty } )}}{{T_{\infty } }},Ec = \frac{{\left( \Omega \right)^{2} }}{{(T_{w} - T_{\infty } )C_{p} }},{\text{Re}}_{r} = \frac{(\Omega r)r}{{2\nu }}, \hfill \\ Sc = \frac{\nu }{{D_{B} }},K_{c} = \frac{{k_{c} C_{\infty }^{2} }}{\Omega },K_{s} = \frac{{k_{s} \sqrt \nu }}{{D_{A} \sqrt \Omega }},S_{\nu } = \frac{{SD_{A} }}{{\sqrt {\Omega \nu } }}, \hfill \\ K_{vs} = S_{\nu } K_{s} = \frac{{Sk_{s} }}{\Omega },\delta = \frac{{D_{B} }}{{D_{A} }} \hfill \\ \end{gathered} $$

Here $k^{*}$ is the permeability parameter, $\lambda$ is the porosity parameter. $F_{r}$ is Forchheimer number, $M$ is the magnetic moment parameter,$N_{b}$ and $N_{t}$ stands for Brownian motion and thermophoresis parameter.$Ec$ is the Eckert number, ${\text{Re}}_{r}$ is the local Reynolds number. $Sc$ is the Schmidt number. $K_{c}$ and $K_{s}$ stand for the homogeneous and heterogeneous reaction parameters respectively. $K_{vs}$ is the surface catalyzed parameter, $\delta$ is the ratio of diffusion coefficients, and $S_{\nu }$ is the interfacial area parameter.

## Numerical procedure

The transformed ordinary differential Eqs. (12–14) and (17) together with boundary conditions (18) are translated into the differential equation of first order. A numerical solution is attained by employing the bvp4c technique by using MATLAB. The newly defined variables are as follows:

20 $$ \begin{gathered} f = Y_{1} ,f^{\prime} = Y_{2} ,f^{\prime\prime} = Y_{3} ,f^{\prime\prime\prime} = yy_{1} ,g = Y_{4} ,g^{\prime} = Y_{5} ,g^{\prime\prime} = yy_{2} , \hfill \\ \theta = Y_{6} ,\theta^{\prime} = Y_{7} ,\theta^{\prime\prime} = yy_{3} , \hfill \\ \phi = Y_{8} ,\phi^{\prime} = Y_{9} ,\phi^{\prime\prime} = yy_{4} . \hfill \\ \end{gathered} $$

Following Eq. (20), the aforementioned set of equations take the following form:

21 $$ yy_{1} = \, \left( \frac{1}{2} \right) \times \left[ {Y_{2}^{2} - Y_{4}^{2} + (M^{2} + \lambda )Y_{2} } \right] - Y_{1} Y_{3} , $$

22 $$ yy_{2} = \left( \frac{1}{2} \right)\left[ {2Y_{2} Y_{4} - 2Y_{5} Y_{1} + (M^{2} - \lambda )Y_{4} } \right], $$

23 $$ yy_{3} = \Pr \left[ { - Y_{7} Y_{1} - N_{b} Y_{7} + N_{t} Y_{7}^{2} - Ec\left( {\frac{1}{{{\text{Re}}_{r} }}Y_{2}^{2} + Y_{5}^{2} + Y_{3}^{2} } \right)} \right], $$

24 $$ yy_{4} \, = Sc\left[ {Y_{8} \left( {1 - Y_{8} } \right)^{2} K_{c} + K_{vs} Y_{8} - Y_{1} Y_{9} } \right] + \frac{{N_{t} }}{{N_{b} }}yy_{3} , $$

25 $$ \begin{gathered} Y_{1} (0) = 0,Y_{2} (0) = \gamma_{1} Y_{3} (0),Y_{4} (0) = 1 + \gamma_{1} Y_{5} (0),Y_{5} (0) = 1 + \gamma_{2} Y_{7} (0), \hfill \\ Y_{9} (0) = K_{s} Y_{8} (0)Y_{2} (\infty ) = 0Y_{4} (\infty ) = 0,Y_{6} (\infty ) = 0,Y_{8} (\infty ) = 1. \hfill \\ \end{gathered} $$

## Drag force coefficient and rate of heat transfer

The Skin friction coefficient and local Nusselt number are given by the following equations:

26 $$ \left\{ \begin{gathered} \left( {{\text{Re}}_{r} } \right)^{1/2} C_{f} = f^{\prime\prime}(0), \hfill \\ \left( {{\text{Re}}_{r} } \right)^{1/2} C_{g} = g^{\prime}(0), \hfill \\ \left( {{\text{Re}}_{r} } \right)^{ - 1/2} Nu = - \theta ^{\prime}(0), \hfill \\ \left( {{\text{Re}}_{r} } \right)^{ - 1/2} Sh = - \phi ^{\prime}(0), \hfill \\ \end{gathered} \right. $$

## Entropy analysis

The volumetric rate of entropy generation in the presence of magnetohydrodynamics with porous media is given by:

27 $$ S^{\prime\prime\prime}_{gen} = \frac{k}{{T_{w}^{2} }}\left[ {\nabla T} \right]^{2} + \frac{\mu }{{T_{w} }}\Psi + \frac{1}{{T_{w} }}\left[ {\left( {J - QV} \right).\left( {E + V \times B} \right)} \right] + \frac{{R_{g} D}}{{C_{\infty } }}\left[ {\nabla C} \right]^{2} + \frac{{R_{g} D}}{{T_{w} }}\left[ {\nabla T.\nabla C} \right], $$

where

28 $$ \Psi = 2\left[ {\left( {u_{r} } \right)^{2} + \frac{1}{{r^{2} }}\left( {v_{\theta } + u} \right)^{2} + w_{z}^{2} } \right] + \left( {v_{z} + \frac{1}{r}w_{\theta } } \right)^{2} + \left( {w_{r} + u_{z} } \right)^{2} + \left[ {\frac{1}{r}u_{\theta } + r\frac{\partial }{\partial r}\left( \frac{v}{r} \right)} \right]^{2} , $$

29 $$ J = \sigma (E + V \times B). $$

Assume that electric field intensity is negligible as compared to the $V \times B$ and $J$ is much greater than $QV$. Under the application of this assumption Eq. (21) takes the following form:

30 $$ \begin{gathered} S^{\prime\prime\prime}_{gen} = \frac{k}{{T_{w}^{2} }}\left[ {\frac{\partial T}{{\partial z}}} \right]^{2} + \frac{\mu }{{T_{w} }}\left( \begin{gathered} 2\left[ {\left( {u_{r} } \right)^{2} + \frac{1}{{r^{2} }}\left( {v_{\theta } + u} \right)^{2} + w_{z}^{2} } \right] + \left( {v_{z} + \frac{1}{r}w_{\theta } } \right)^{2} + \left( {w_{r} + u_{z} } \right)^{2} \hfill \\ + \left[ {\frac{1}{r}u_{\theta } + r\frac{\partial }{\partial r}\left( \frac{v}{r} \right)} \right]^{2} \hfill \\ \end{gathered} \right) \hfill \\ + \frac{{\sigma_{o}^{2} }}{{T_{w} }}\left[ {u^{2} + v^{2} } \right] + \frac{{R_{g} D}}{{C_{\infty } }}\left[ {\frac{{\partial C_{a} }}{\partial z}} \right]^{2} + \frac{{R_{g} D}}{{T_{w} }}\left[ {\frac{{\partial C_{a} }}{\partial z}.\frac{\partial T}{{\partial z}}} \right], \hfill \\ \end{gathered} $$

where $S^{\prime\prime\prime}_{gen}$ is the actual entropy generation rate. On applying the similarity transformation, we have

31 $$ N_{G} = \alpha \theta^{{\prime}{2}} + Br\left( {\frac{12}{{\text{Re}}}\left\{ {f^{{\prime}{2}} + 2R^{2} \left\{ {f^{{\prime\prime}{2}} + g^{{\prime}{2}} } \right\}} \right\}} \right) + M^{2} \left( {f^{{\prime}{2}} + g^{2} } \right) + {\text{Re}} \sum \left( {\phi^{{\prime}{2}} + \frac{{\phi^{\prime}\theta^{\prime}}}{\alpha }} \right), $$

Here,$N_{G}$ is the entropy generation number,$\alpha = \frac{\Delta T}{{T_{\infty } }}$ is the dimensionless temperature difference, $R = \frac{r}{l}$ is the radius dimensionless radius. $\Sigma = \frac{{R_{g} DC_{\infty } }}{k}$ is the diffusion constant parameter. ${\text{Re}} = \frac{{\Omega l^{2} }}{\nu }$ is the rotational Reynolds number, $Br = \frac{{\mu \Omega^{2} l^{2} }}{k}$ is the Brinkman number.

## Results and discussion

**Figure 2:**
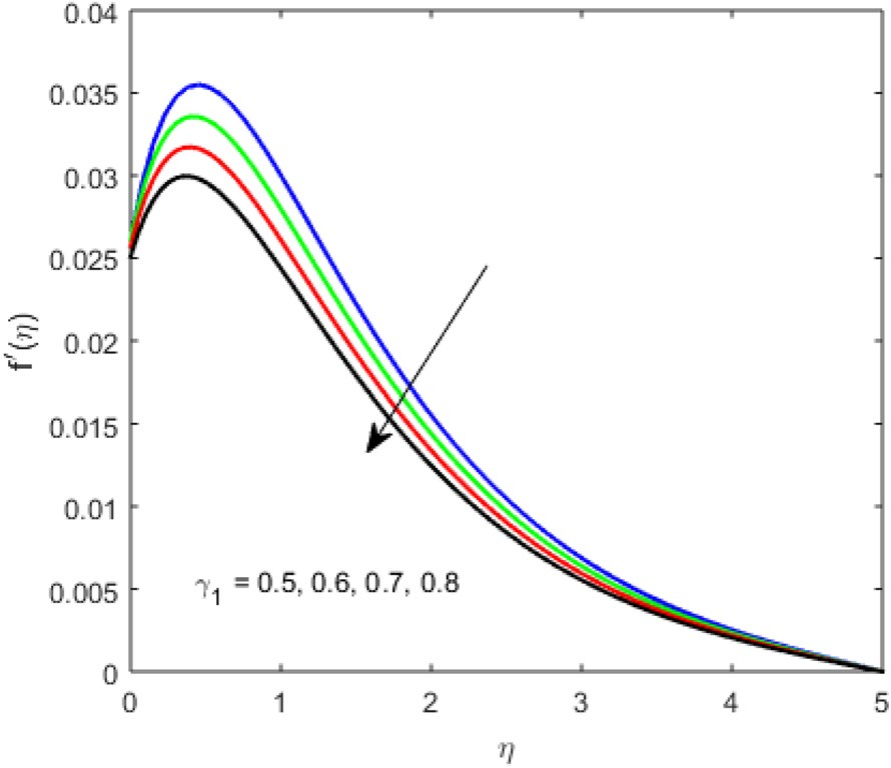
Velocity distribution for varying $\gamma_{1}$.

**Figure 3:**
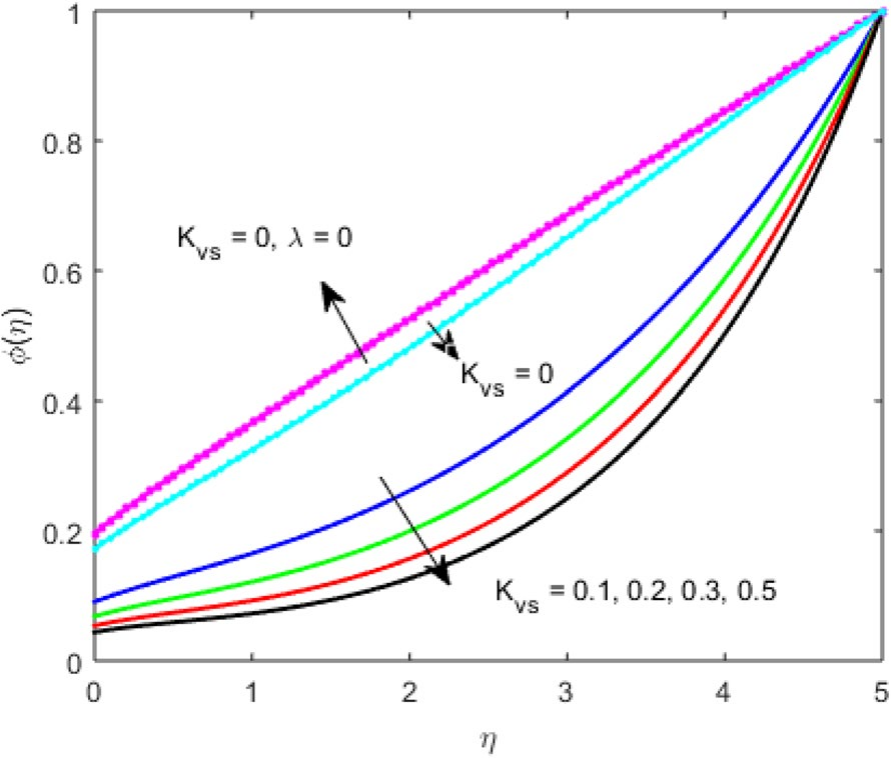
Concentration distribution for varying $K_{vs}$.

**Figure 4:**
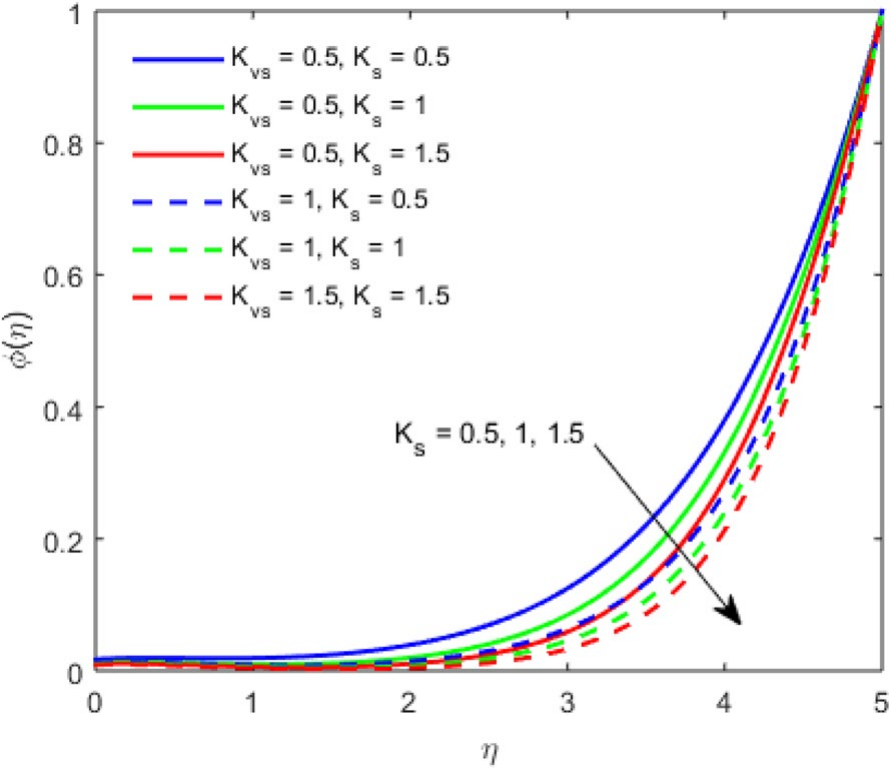
Concentration distribution for varying $K_{s}$.

**Figure 5:**
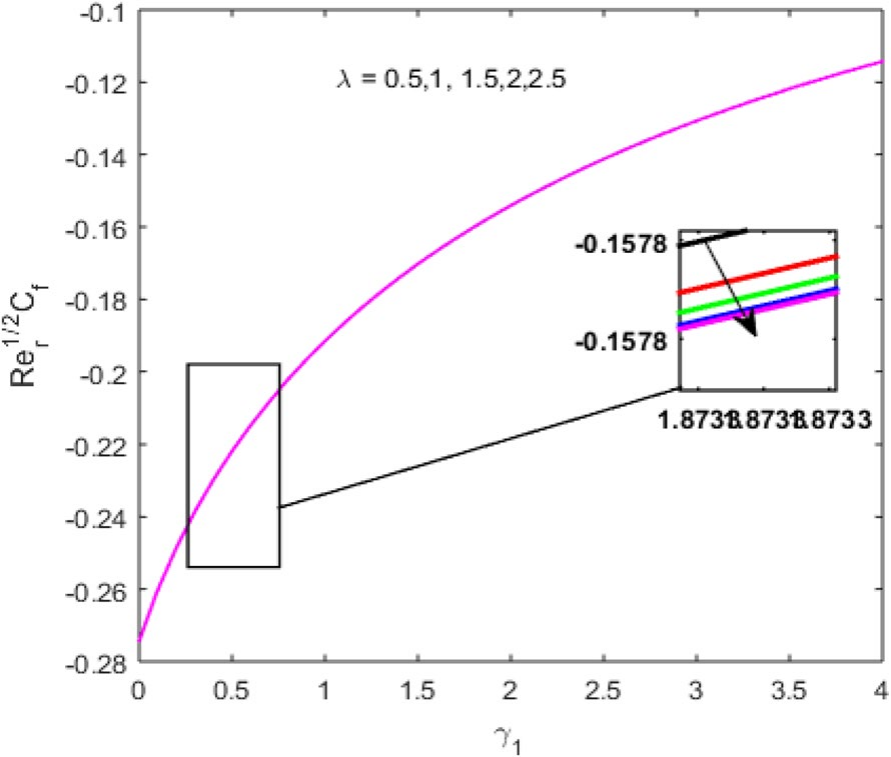
$\left( {{\text{Re}}_{r} } \right)^{1/2} C_{f}$ versus $\gamma_{1}$ for varying $\lambda$.

**Figure 6:**
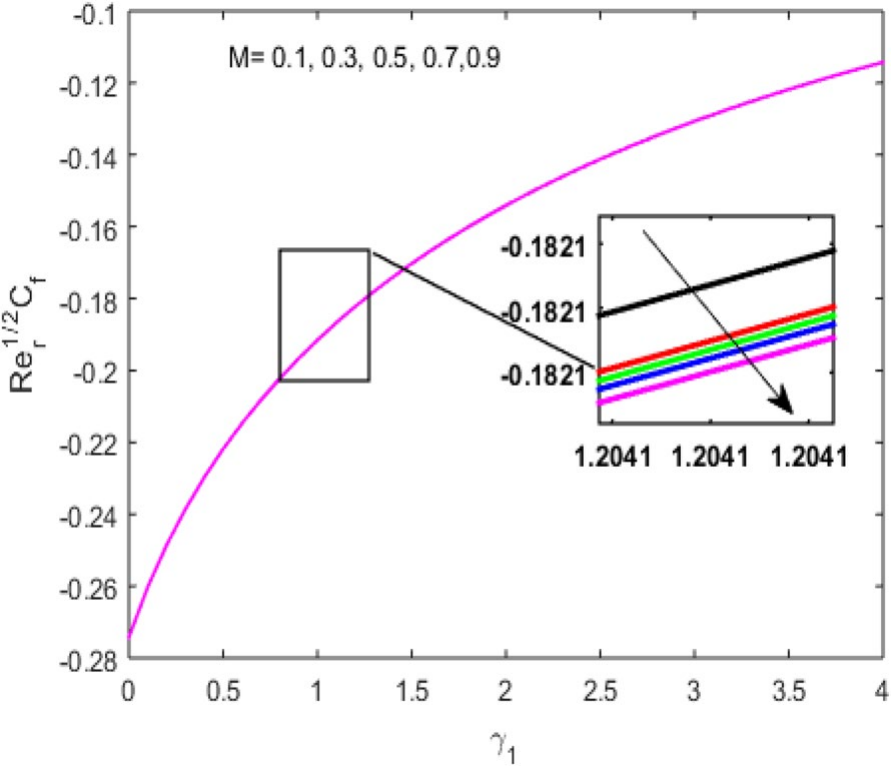
$\left( {{\text{Re}}_{r} } \right)^{1/2} C_{f}$ versus $\gamma_{1}$ for varying $M$.

**Figure 7:**
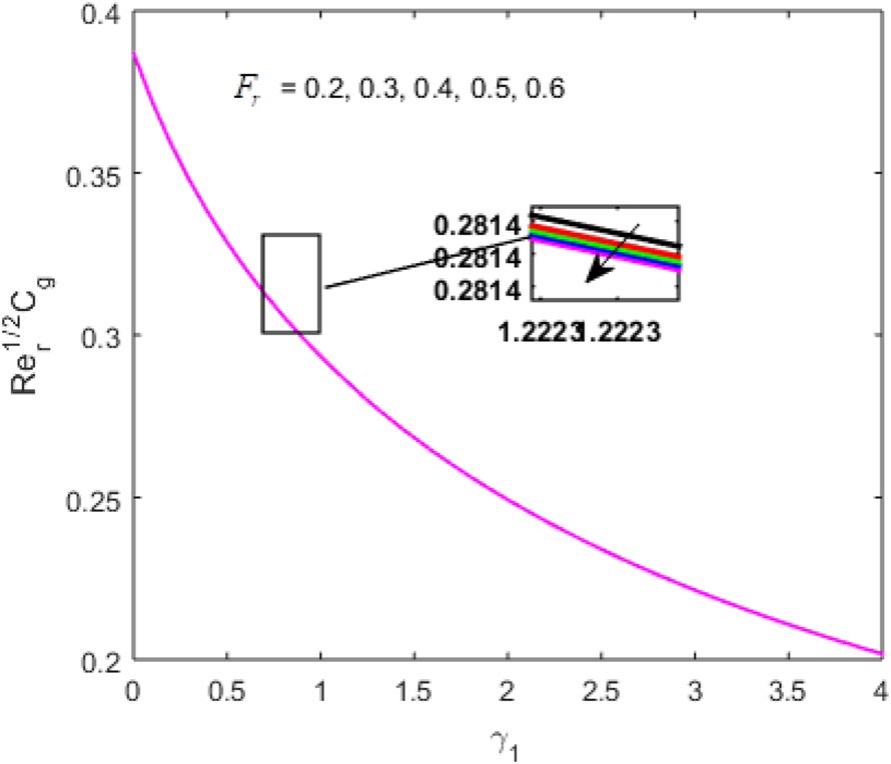
$\left( {{\text{Re}}_{r} } \right)^{1/2} C_{g}$ versus $\gamma_{1}$ for varying $F_{r}$.

**Figure 8:**
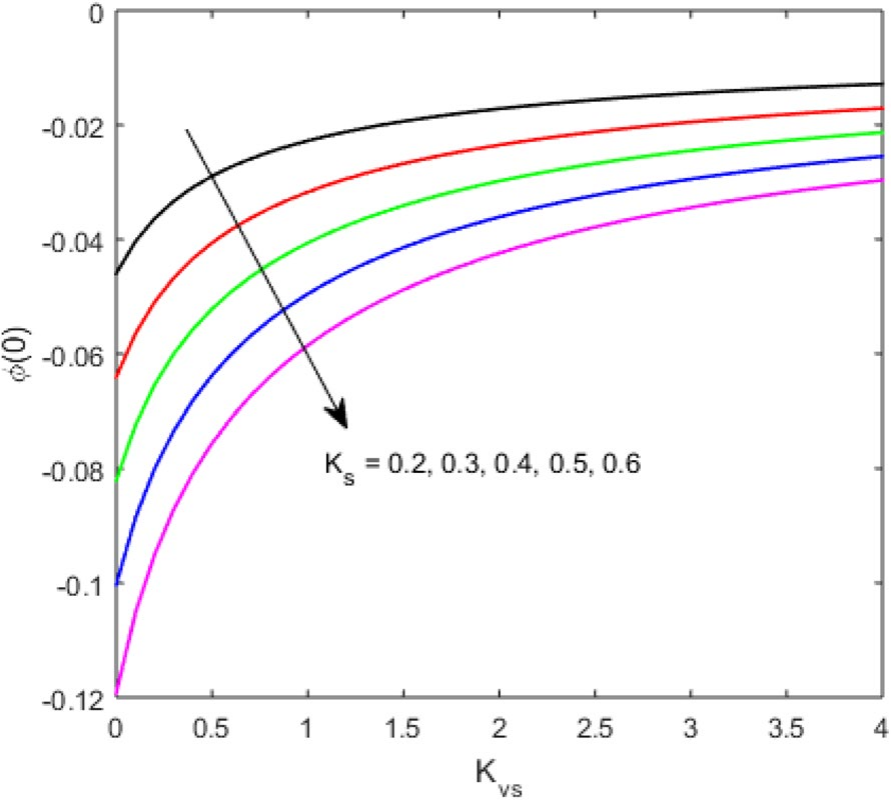
Concentration distribution versus $K_{vs}$ for varying $K_{s}$.

**Figure 9:**
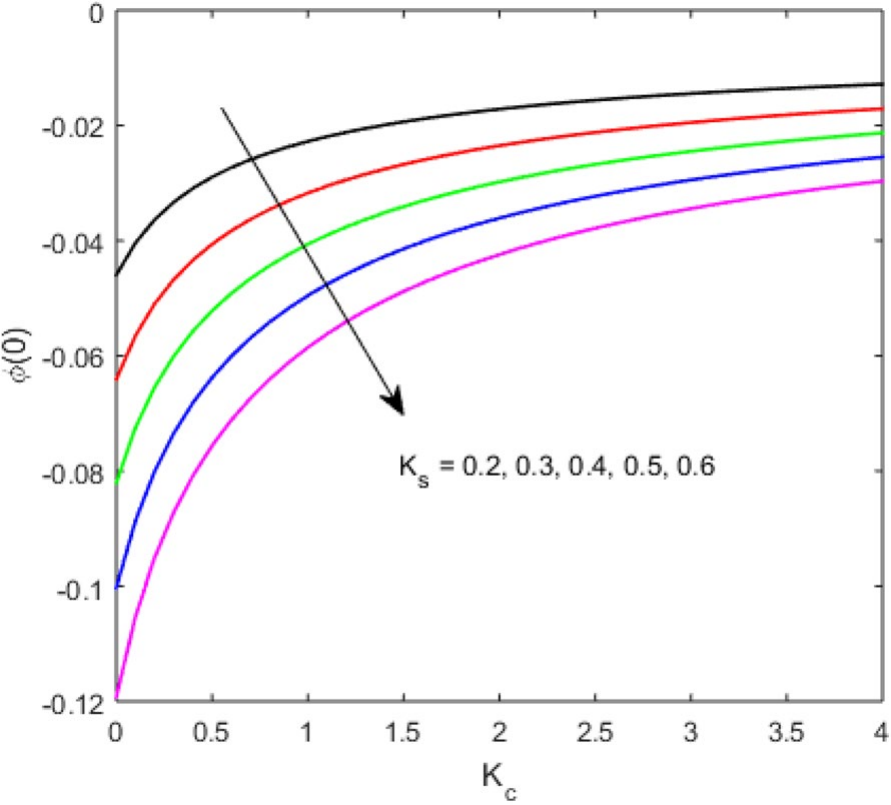
Concentration distribution versus $K_{c}$ for varying $K_{s}$.

**Figure 10:**
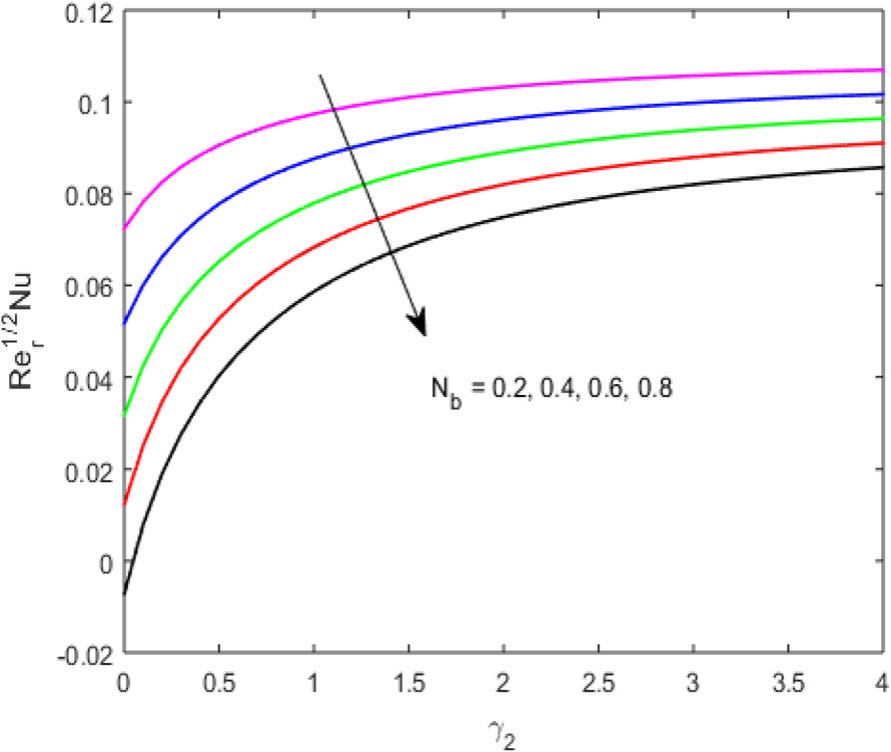
$\left( {{\text{Re}}_{r} } \right)^{ - 1/2} Nu$ versus $\gamma_{2}$ for varying $N_{b}$.

**Figure 11:**
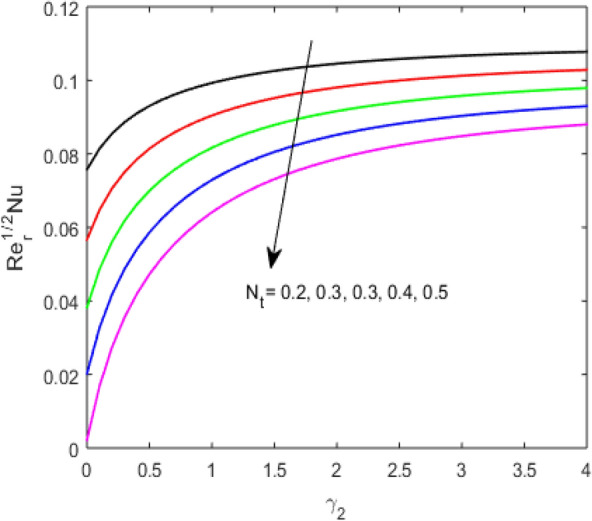
$\left( {{\text{Re}}_{r} } \right)^{ - 1/2} Nu$ versus $\gamma_{2}$ for varying $N_{t}$.

**Figure 12:**
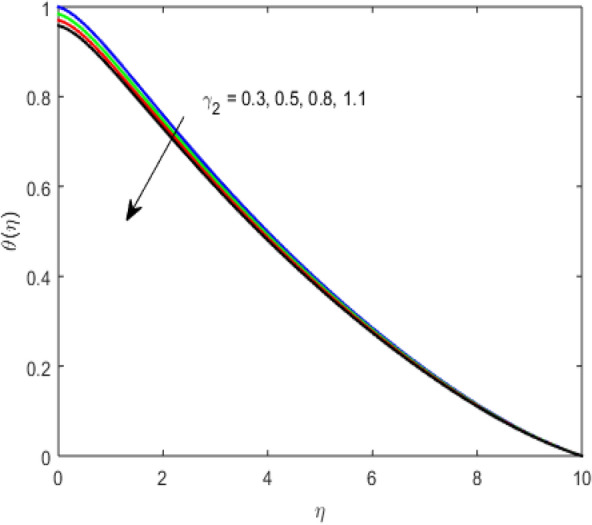
Temperature distribution for varying $\gamma_{2}$.

**Figure 13:**
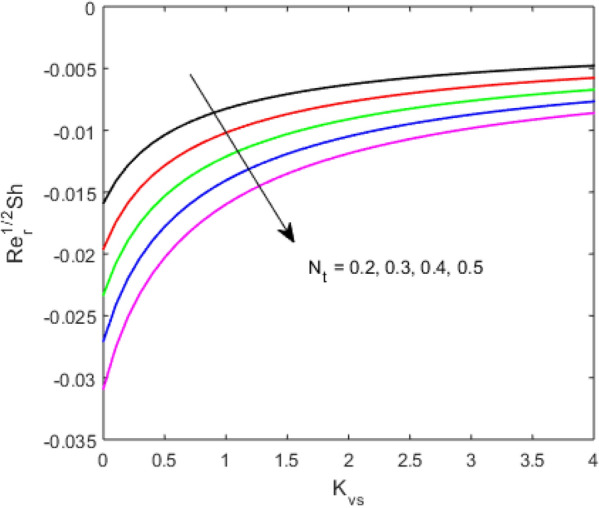
Sherwood number $\left( {{\text{Re}}_{r} } \right)^{ - 1/2} Sh$ versus $K_{vs}$ for varing $N_{t}$.

**Figure 14:**
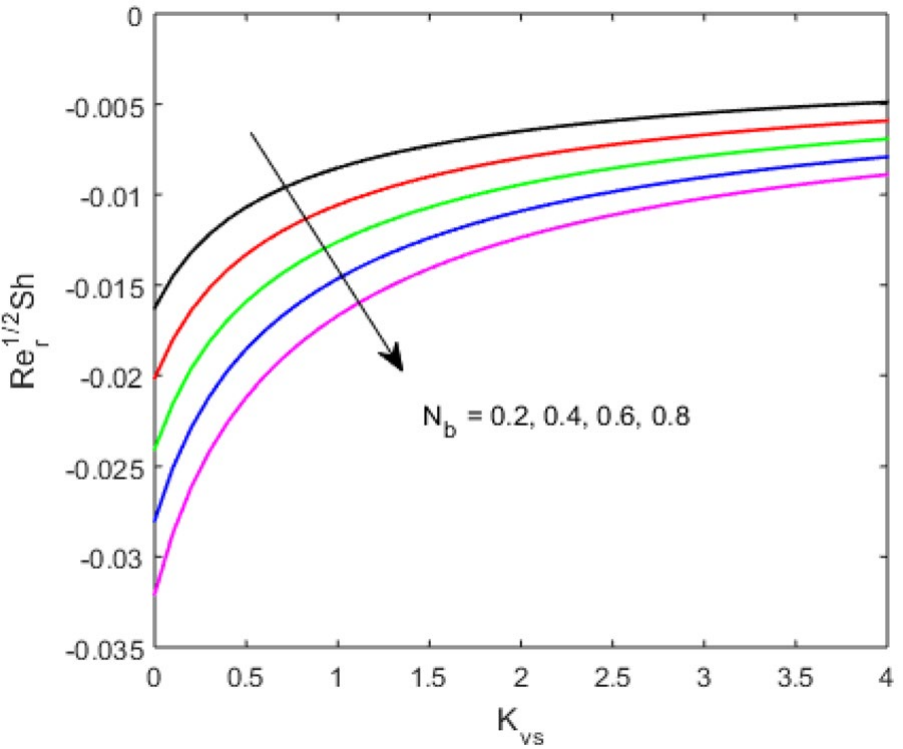
Sherwood number $\left( {{\text{Re}}_{r} } \right)^{ - 1/2} Sh$ versus $K_{vs}$ for varing $N_{b}$.

**Figure 15:**
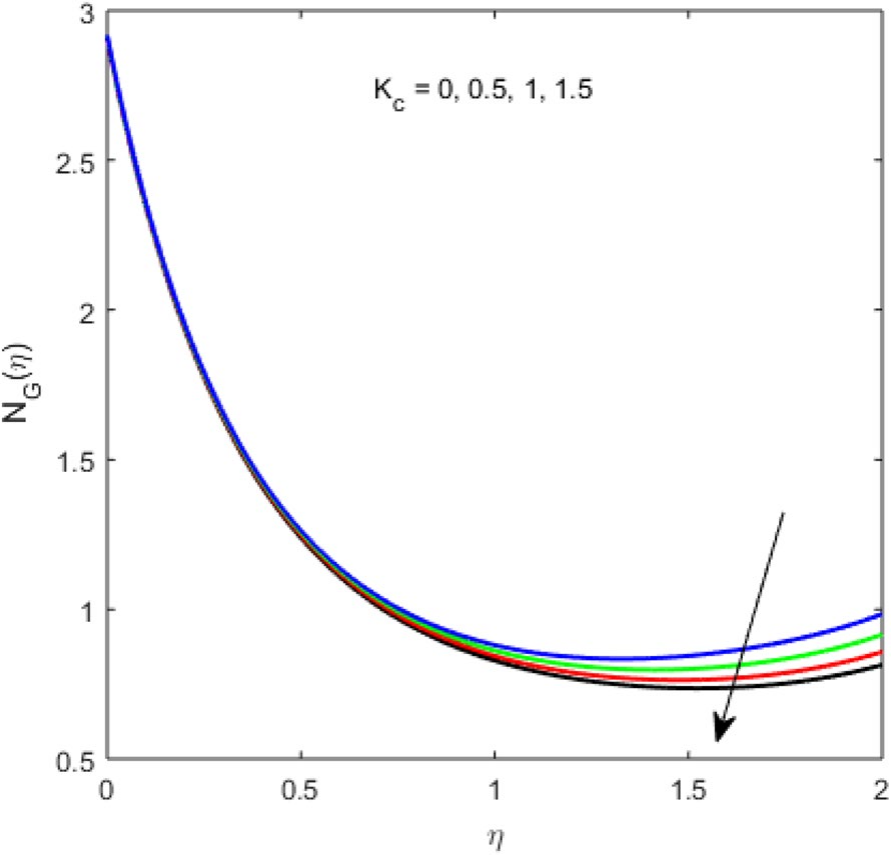
Entropy generation variation for increasing $K_{c}$.

**Figure 16:**
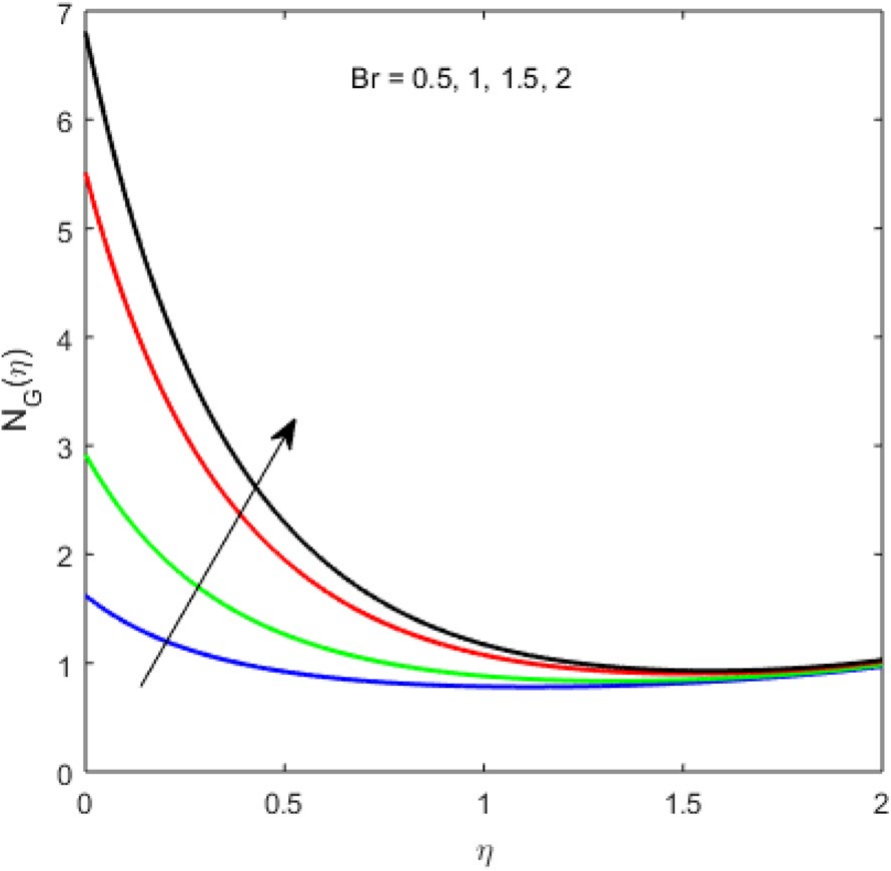
Entropy generation variation for increasing $Br$.

**Figure 17:**
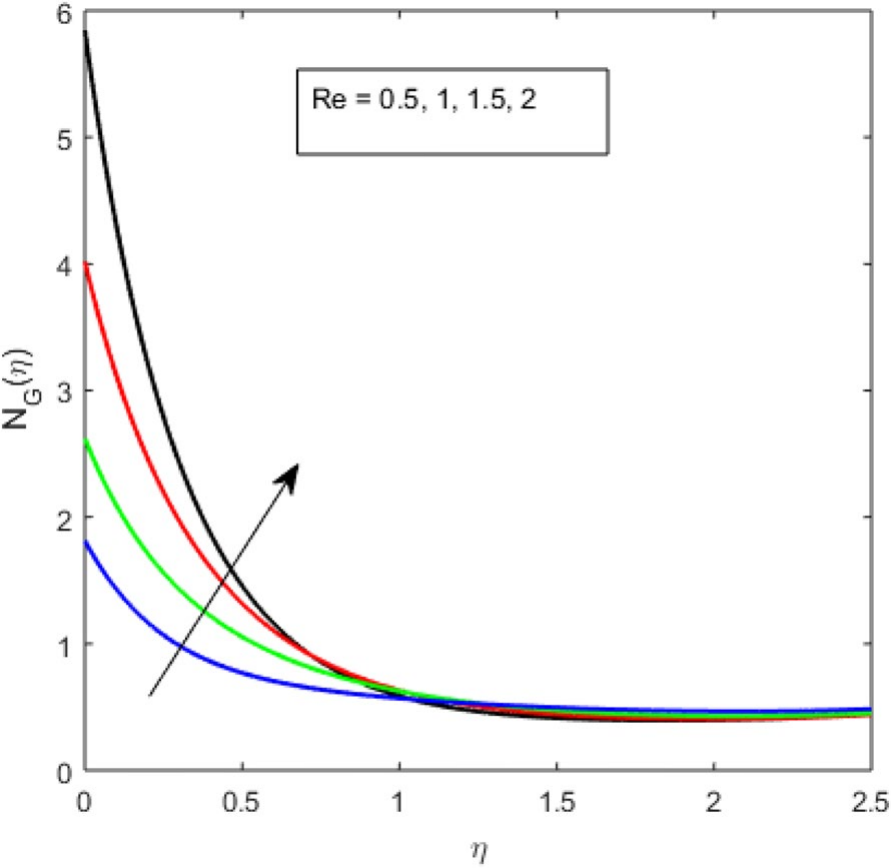
Entropy generation variation for increasing ${\text{Re}}$.

The present section examines the solution diversities for varying pertinent parameters graphically. In Fig. 2, the representation of velocity distribution is shown for different values of the velocity slip parameter $\gamma_{1}$. The velocity profile at a far-off distance from the disk is not affected by the variation by the slip parameter. However, near the disk, the amplitude of the velocity profile is maximum for the smallest value of the velocity slip parameter. It happens because as the value of the velocity slip parameter increases, fluid layers would experience less drag force. Figure 3 represents declining concentration distribution for a higher estimation of the $K_{vs}$. Physically, the value of surface catalyzed parameter speeds up the reaction rate with increasing reaction interface for porous medium. The case of non-catalytic porous media $\left( {\lambda = 1,K_{vs} = 0} \right)$ indicates that the concentration profile decreases. It is noted that in the absence of porous media $\lambda = 0$ and with a non-catalytic surface, the concentration is maximum. Figure 4 describes the decline in the concentration curve for the consumption of reactant species. It has been visualized that the presence of surface catalyzed reaction promotes the consumption of reactant species. Figure 5 represents the coefficient of drag force versus the velocity slip parameter for escalating values of the porosity parameter. The graphical analysis of the coefficient of drag force indicates that the negligible decline occurs for increasing the porosity parameter. Surface catalysis on porous medium enhances the resistive forces which reduce the fluid motion. At the maximum value of the slip parameter, the coefficient of drag force is maximum. Figure 6 is drawn for the coefficient of drag force versus velocity slip parameter for increasing the magnetic moment parameter. It is witnessed that the coefficient of drag force in radial direction declines negligibly as the magnetic moment parameter increase because the presence of the magnetic field decreases the momentum boundary layer thickness due to Lorentz force. Figure 7 is the graphical depiction of coefficients of drag force in tangential direction versus velocity slip parameter for increasing Forchheimer number. Physically increasing slip parameter and inertia coefficient causes the decrease in momentum boundary layer thickness along with velocity profiles. Increasing $F_{r}$ means the increase in inertial coefficients resulting in a decline in drag force coefficients. Figure 8 signifies the variation of wall concentration versus $K_{vs}$ for increasing heterogeneous reaction parameter $K_{s}$ and. A declining trend is observed in wall concentration. Figure 9 shows that by keeping $\eta$ fixed and increasing the value of the HET parameter, the concentration profiles versus HOM parameter declines. As the reaction proceeds and the reactants consume causes a decline in the concentration distribution at the wall. Figures 10 and 11 exhibit that the heat transfer rate versus thermal slip parameter for escalating values of the Brownian diffusion coefficient and thermophoresis parameter. Physically, increasing the Brownian motion parameter causes the irregular movements of nanoparticle which enhances the collision among particles. An increase in thermophoresis parameter value causes a thicker thermal boundary layer. Therefore, by increasing $N_{t}$ results the movement of particles from the region of higher temperature to the region of lower temperature. Figure 12 shows the thermal profile for increasing the value of the thermal slip parameter. The decline in thermal profile points to the fact of a decrease in heat transfer rate due to the thermal slip parameter from the surface to the adjacent fluid layers. Figures 13 and 14 show the graphical trends of mass transfer rate versus increasing surface catalyzed parameter $K_{vs}$ for escalating values of $N_{b}$ and $N_{t}$. The decline in mass transfer rate for thermophoresis and the Brownian motion parameter is witnessed. Figure 15 portrays the graphical sketch of the rate of disorder for increasing homogeneous reaction parameter. The increasing trend for increasing the HOM reaction parameter $K_{c}$ is elaborated in Fig. 15. The physical aspect behind the fact of decreasing the rate of a disorder by increasing $K_{c}$, is directly linked to temperature. Figure 16 reflects the increase in the degree of the disorder by escalating Brinkman number $Br$ leads to the fact that conducting particles transport the heat and viscous dissipation prevails throughout. Increasing $Br$ means that more viscous heating relative to conductive heat transfer which causes the production of additional heat. It causes a disturbance in the entire network and a large amount of heat is produced among the layers of the moving fluid. Hence, the Brinkman number has a direct impact on the entropy generation process. Figure 17 reveals that upon increasing $Re$ the degree of disorder decrease heat transfer irreversibility is more than viscous dissipation irreversibility, which means that for higher estimation of $Re$ causes the decline in entropy generation rate.

Table 2 is erected to corroborate our presented mathematical model by comparing the numerous values of $\lambda$ with Kameswaran et al.^51^ and Hayat et al.^36^ by considering $\gamma_{1} = 0.$

**Table 2:** Numerical results of $f^{\prime\prime}(0)$ obtained for increasing $\lambda$ by taking $\gamma_{1} = 0$.

$\lambda$	Kameswaran et al.^51^		Hayat et al.^36^		Our results
	Analytical	Numerical	Analytical	Numerical	Bvp4c
1	1.41421356	1.41421356	1.4142	1.4142	1.41422
1.5	1.581138833	1.58113883	1.5811	1.5811	1.58113
2	1.73205081	1.73205081	1.3720	1.7321	1.73215
5	2.44948974	2.44948974	2.4494	2.4495	2.44959

## Conclusion

In this present study, we have emphasized a Darcy–Forchheimer nanofluid flow in permeable media over the rotating disk with velocity and thermal partial slip. HOM–HET isothermal cubic autocatalysis is employed with homogeneous porous media. Earlier published studies considered wall surface. Nevertheless, in this assumption, stretched rotating surfaces and porous media consist of the same catalyst. A numerical solution to the modeled problem is obtained. The key observations of the existing study are as follows:

- The HET catalysis in porous media causes a fast reaction rate and slow reaction time. The presence of the HET catalysis in a permeable media is being discussed first time. No such investigation deliberated it.
- Wall concentration is greatly influenced by the surface catalyzed reaction rate. This effect is unique as no other research has discussed this effect.
- Upon increasing the strength of the HOM–HET reaction, the concentration near the surface of porous media decreases.
- The rate of the disorder is meager for increasing the HOM reaction parameter.
- The velocity profile near the disk is immensely influenced by the slip parameter and its effect gradually diminishes as we move away from the disk.
- The Skin friction coefficients versus the velocity slip parameter along both axes are on the decline for growing estimates of porosity parameter and Forchheimer number.
- The concentration distribution is declined for a higher estimation of the surface catalyzed parameter.
